# Genome-wide association analysis of insomnia using data from Partners Biobank

**DOI:** 10.1038/s41598-020-63792-0

**Published:** 2020-04-24

**Authors:** Wenyu Song, John Torous, Joe Kossowsky, Chia-Yen Chen, Hailiang Huang, Adam Wright

**Affiliations:** 1 Departmet of Medicine, Brigham and Women’s Hospital, Harvard Medical School, Boston, USA; 2 Department of Biomedical Informatics, Harvard Medical School, Boston, USA; 3 Department of Psychiatry, Beth Israel Deaconess Medical Center, Harvard Medical School, Boston, USA; 4 Department of Anesthesiology, Critical Care & Pain Medicine, Boston Children’s Hospital, Harvard Medical School, Boston, USA; 5 Division of Clinical Psychology and Psychotherapy, University of Basel, Basel, Switzerland; 6 Psychiatric and Neurodevelopmental Genetics Unit, Analytic and Translational Genetics Unit, Massachusetts General Hospital, Harvard Medical School, Boston, USA; 7 Stanley Center for Psychiatric Research, Broad Institute of MIT and Harvard, Boston, USA; 8 Department of Biomedical Informatics, Vanderbilt University Medical Center, Nashville, USA; 9 Partners eCare, Partners HealthCare, Boston, USA

**Keywords:** Genome informatics, Psychiatric disorders

## Abstract

Insomnia is one of the most prevalent and burdensome mental disorders worldwide, affecting between 10–20% of adults and up to 48% of the geriatric population. It is further associated with substance usage and dependence, as well other psychiatric disorders. In this study, we combined electronic health record (EHR) derived phenotypes and genotype information to conduct a genome wide analysis of insomnia in a 18,055 patient cohort. Diagnostic codes were used to identify 3,135 patients with insomnia. Our genome-wide association study (GWAS) identified one novel genomic risk locus on chromosome 8 (lead SNP rs17052966, p = 4.53 × 10^−9^, odds ratio = 1.28, se = 0.04). The heritability analysis indicated that common SNPs accounts for 7% (se = 0.02, p = 0.015) of phenotypic variation. We further conducted a large-scale meta-analysis of our results and summary statistics of two recent insomnia GWAS and 13 significant loci were identified. The genetic correlation analysis yielded a strong positive genetic correlation between insomnia and alcohol use (rG = 0.56, se = 0.14, p < 0.001), nicotine use (rG = 0.50, se = 0.12, p < 0.001) and opioid use (rG = 0.43, se = 0.18, p = 0.02) disorders, suggesting a significant common genetic risk factors between insomnia and substance use.

## Introduction

Insomnia is a highly prevalent sleep disorder characterized by the inability to fall asleep or maintain sleep^1^ and affects 10–20% of the adult population^2,3^. It is characterized by heterogeneous phenotypes and equifinality, which might reflect different underlying causal mechanisms^4^, including life style, stress and molecular mechanisms (for a review, see^5^). It is commonly comorbid with other physical and psychiatric disorders^6,7^.

Genetic contributions to insomnia have been demonstrated in both family and twin studies with the reported heritability being estimated at 25–45%^8^. Candidate gene studies have highlighted genetic variants in numerous systems including the circadian gene *CLOCK*^9^, the GABAergic system^10^, the adenosinergic system^11^, and the serotonergic system^12^.

A number of genome-wide association studies (GWAS) have been conducted examining the insomnia phenotype. In two recent studies, large-scale cohorts were developed using data from UK Biobank and the combination of UK Biobank and 23andMe yielding 57 and 202 significant loci, respectively^13,14^. Another study using survey data of soldiers in the Army Study To Assess Risk and Resilience in Servicemembers (STARRS) study identified one significant locus^15^. These studies also identified genetic correlations between insomnia and various clinical conditions, such as schizophrenia, type 2 diabetes, and depression^13,15^. Other studies have identified several insomnia related genes, such as *CACNA1C*^16^, *RBFOX3*^17^, *PAX8*^18^ and *MEIS1*^19^.

In most previous studies, insomnia phenotypes were assessed through self-report, which could miss useful information and reflect only part of disorder status. Since insomnia can be a chronic process with different trajectories and multiple complications in clinical settings, it is important to conduct studies specifically targeting clinical patient populations^20^. Because of complex underlying mechanisms of insomnia and its various clinical manifestations, obtaining a clinically well-defined subject cohort is critical for genetic association analysis. Electronic health records (EHRs) from large medical institutes comprise a uniquely valuable data source to help identify genetic associations within very specific clinical conditions^21^.

In this study, we utilized a large-scale clinical database to explore the genetic underpinnings of insomnia and calculated the genetic correlation between insomnia and various clinical conditions. Further, we conducted a meta-analysis of our results combined with recent insomnia GWAS to discover novel genomic loci.

## Methods

### Clinical database

All the clinical data and genetic data in this study were obtained from the Partners Biobank^22^. The Partners Biobank is a large integrated database which contains clinical data from Partners HealthCare for approximately 90,000 consented patients, and genomic data for approximately 25,000 of them. The clinical data including patient family history, demographic information, diagnosis, medication records, lab test results and clinical notes. The clinical data is derived from the electronic health records, which have been collecting patient data since 1990. The informed consent was obtained from all study participants and/or their legal guardians. The study’s protocol was reviewed and approved by Partners Human Research Committee. All methods were performed in accordance with the relevant guidelines and regulations.

### Electronic health record-derived phenotypes

We generated an ICD 9 and ICD10 code list for insomnia, three major substance use disorders and a series of relevant clinical conditions, including multiple psychiatric disorders and type 2 diabetes, then used these codes to identify our case cohort (Supplementary Table 1).

### The ICD codes of insomnia include the following definitions

307.4*: specific disorders of sleep of nonorganic origin; 327.0*: organic disorders of initiating and maintaining sleep; 780.51: insomnia with sleep apnea, unspecified; 780.52: insomnia, unspecified; G47.0*: insomnia; F51.0*: insomnia not due to a substance or known physiological condition.

We reviewed 15,750,104 diagnosis records, which were collected between 1991 and 2018, to identify patients meeting our insomnia phenotype definition. The control cohort consisted of patients not meeting the insomnia phenotype, and also excluded patients with any other kind of sleep disorders, including snoring, periodic limb movement, sleep related leg cramps, sleep related bruxism and hypersomnia.

For the three substance use disorders, the case cohort included patients with at least one corresponding ICD code of substance dependence, substance abuse or long-term substance use disorder. The control group consisted of 12,205 patients without any record of substance use disorder (nicotine, alcohol, opioid, cannabis, cocaine and amphetamine).

### Genotyping, imputation and quality control

The genotyping was performed by Partners Biobank using the Illumina Multi-Ethnic Global (MEG) array (Illumina, Inc., San Diego, CA) including 1,779,763 SNPs. Prior to imputation, QC steps were conducted, including: a. sample-level filtration: any samples with a discrepancy between the reported and predicted sex were removed. b. SNP-level filtration: removal of sites with invalid alleles, duplicate, monomorphic, indel, allele mismatch, low call rate (less than 90%). The SNPs that were not in the reference panel were also removed. The imputation was performed using the Michigan Imputation Server with Minimac3^23^. The HRC (Version r1.1 2016) reference panel consisting of 64,940 haplotypes of predominantly European ancestry was used^24^.

Post-imputation quality control was conducted to select high-quality SNPs and control for population stratification. In all analyses, only autosomal biallelic SNPs with minor allele frequencies (MAF) of at least 1%, an info score above 0.8 and call rates above 98% were retained, which led to 5,508,534 SNPs. The present analysis included only individuals of European ancestry, which were reported by patients, to minimize the risk for confounding due to ancestry differences. A principal components analysis (PCA) was applied to characterize population structure.

### Statistical analysis

PLINK 1.90 was used to conduct the genome-wide association analysis, adjusted for age, sex and the top 10 principal components^25^.The Genome-based restricted maximum likelihood (GREML) method implemented in GCTA was used to estimate the percentage of variance explained by common SNPs and calculate the genetic correlations^26,27^. LD Score Regression (LDSC) was used to calculate the genetic correlations between our results and publicly available GWAS studies^28^. FUMA and MAGMA were used to conduct the gene-based test and pathway enrichment analysis^29^. METAL was used for the meta-analysis between our results and published insomnia GWAS^30^.

A standard genome-wide significance threshold of p < 5 × 10^−8^ was chosen for SNP identification and r^2^ = 0.6 was set as the cutoff to define LD block. All phenotyping analyses were conducted using R (version 3.3.3).

## Results

We used diagnostic data from Partners Biobank to identify cases with insomnia and controls. The study cohort comprised of 21,310 patients of European ancestry with 11,420 females (53.6%) and 9,890 males (46.4%). The mean age was 59.7 (SD = 16.70). From a total of 15,750,104 patient visit records, we generated an ICD9/ICD10 list for the insomnia phenotype. The diagnosis definition for the cases included primary insomnia, insomnia due to medical conditions and insomnia due to psychiatric disorders. We removed patients with documented comorbid sleep disorder symptoms, including snoring, periodic limb movement, sleep related leg cramps, sleep-related bruxism and hypersomnia. Using this list, we obtained 3,135 case subjects. The control group consisted of 14,920 patients without any record of insomnia or other sleep disorder symptoms.

**Figure 1:**
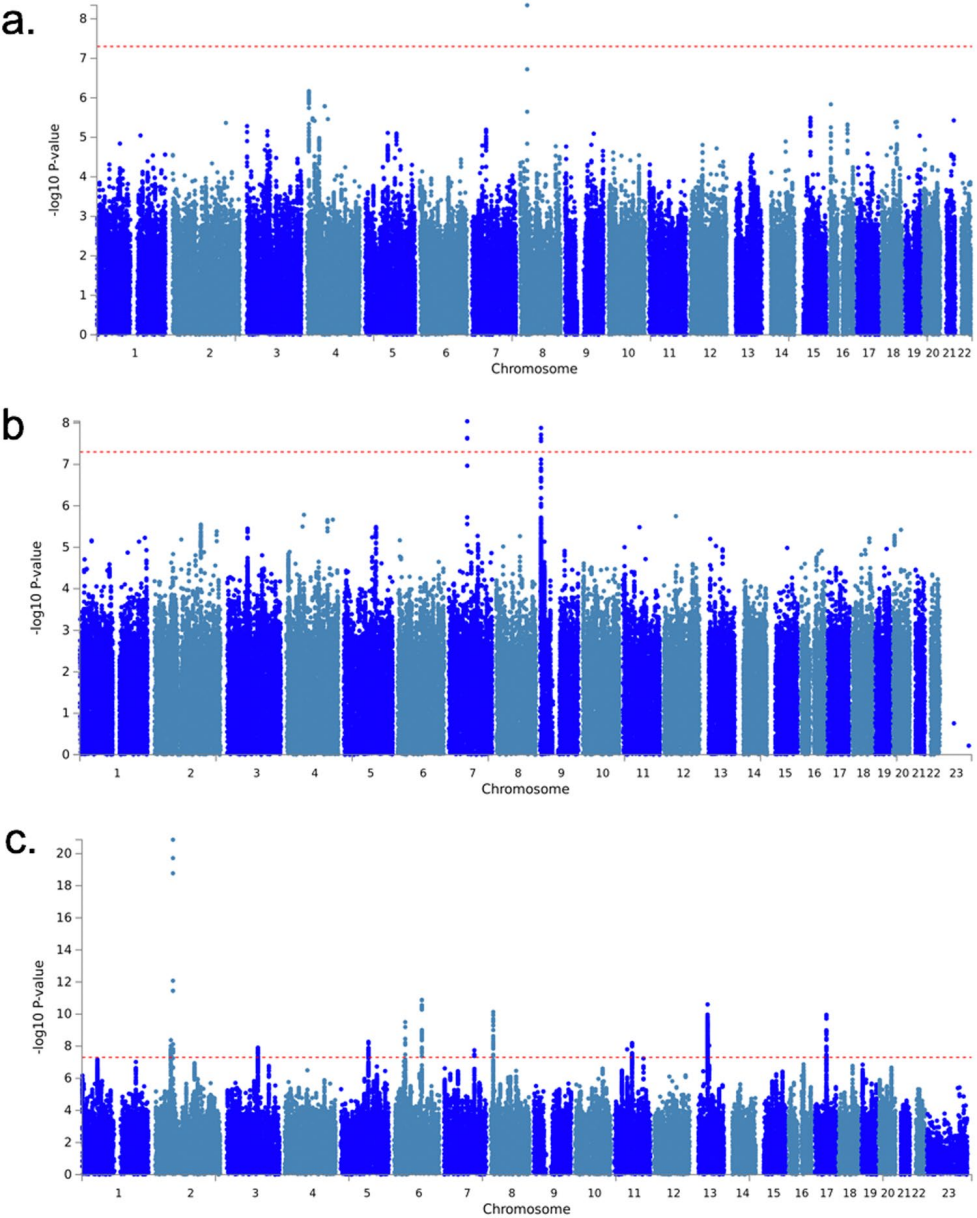
Manhattan plot for Insomnia. (**a)** EHR based phenotype **(b)**. Meta-analysis 1 (**c)**. Meta-analysis 2.

Using high-quality imputed SNPs, a genome wide association analysis was conducted for the insomnia phenotype. Setting the p-value threshold at 5 × 10^−8^, one novel genomic risk locus was identified on chromosome 8p21.2 (Fig. 1a, Supplementary Fig. 1a, Genomic Inflation Factor λ 1.007). The leading SNP was rs17052966 (p = 4.53 × 10^−9^) (Table 1), located inside the gene region of the long non-coding RNA (lncRNA), *CTD-*2*168K21.1*. Using FUMA (Functional Mapping and Annotation) and MAGMA (Multi-marker Analysis of GenoMic Annotation) pipeline, 8 protein-coding genes were identified in the 10 kb distance window, including *LOXL2, ENTPD4, ADAMDEC1, ADAM7, NEFM, EBF2, BNIP3L* and *ADRA1A*. Previous research has linked these genes to sleep related disorders, psychiatric disorders and neurodegenerative disorders (Table 2)^31–38^. 27 other SNPs reached suggestive threshold (5 × 10^−6^) were also identified (Table 1). Among them, multiple SNPs on Chromosome 4 were close to gene *SORCS2*, which functions as a receptor for the precursor form of neurotrophin^39^.

**Table 1:** Summary of variants associated with insomnia.

**Chr**	**SNP**	**BP**	**A1**	**A2**	**OR**	**p-value**	**Genes**
2	rs10177310	192369816	T	C	1.53	4.32E-06	*MYO1B*
4	rs11723611	7218349	G	C	0.84	7.60E-07	*SORCS2*
4	rs28584625	7218452	T	C	0.84	7.05E-07	*SORCS2*
4	rs4689647	7221633	C	T	0.84	7.82E-07	*SORCS2*
4	rs4689654	7223348	T	C	0.84	9.57E-07	*SORCS2*
4	rs11728437	7223543	C	T	0.84	1.07E-06	*SORCS2*
4	rs11728393	7223566	G	A	0.84	1.04E-06	*SORCS2*
4	rs11724245	7223787	A	C	0.84	7.37E-07	*SORCS2*
4	rs11728465	7223793	G	A	0.84	7.34E-07	*SORCS2*
4	rs4689655	7224033	C	T	0.84	7.26E-07	*SORCS2*
4	rs73255313	20622647	G	A	1.18	3.65E-06	*SLIT2*
4	rs73240203	20622681	C	T	1.19	3.30E-06	*SLIT2*
4	rs7663406	27782185	T	G	1.15	3.83E-06	*AC007106.1*
4	rs72641560	64369909	C	A	0.77	1.63E-06	*RP11-12K22.1*
4	rs113864068	64375089	G	A	0.77	1.65E-06	*RP11-12K22.1*
4	rs147927217	75722919	A	G	1.82	3.47E-06	*BTC*
8	rs117915572	24848905	T	C	1.29	1.90E-07	*CTD-2168K21.1*
**8**	**rs17052966**	**24857168**	**T**	**C**	**1.28**	**4.53E-09**	***CTD-2168K21.1***
8	rs2950347	24862709	T	G	1.19	2.25E-06	*CTD-2168K21.1*
15	rs9944172	44769215	G	A	1.25	3.85E-06	*CTDSPL2*
15	rs4611428	44815160	A	G	1.26	4.03E-06	*CTDSPL2*
15	rs2556563	44826477	T	C	1.26	3.20E-06	*EIF3J-AS1*
15	rs2556565	44844262	A	G	1.25	4.82E-06	*EIF3J*
16	rs13380453	6201922	A	G	0.87	1.46E-06	*RP11-420N3.2:RBFOX1*
16	rs233534	66293829	A	G	0.82	4.71E-06	*RNA5SP428*
18	rs77325800	51183914	A	T	1.21	4.15E-06	*RPL29P32*
18	rs6566994	56303425	C	A	1.14	4.06E-06	*RPL9P31*
21	rs73227948	42213747	A	G	1.24	3.74E-06	*DSCAM*
*2*	*rs113851554*	66750564	*T*	*G*	*1.14*	*9.60E-03*	*MEIS1*
5	*rs701394*	80296487	*G*	*A*	*1.07*	*9.70E-03*	*RASGRF2*
5	*rs12187443*	102660400	*C*	*T*	*0.91*	*5.90E-03*	*PAM*
7	*rs8180817*	114047542	*C*	*G*	*0*.9*0*	*3.50E-04*	*FOXP2*
*9*	*rs7044885*	81739348	*C*	*G*	*0.99*	*7.31E-04*	*RP11-165H23.1*

GWAS results for insomnia at significance p < 5x10^−8^ (bold) and suggestive results p < 5 × 10^−6^. SNPs in italic in last five rows were identified as significant SNPs for sleep disorder in previous studies. OR: odds ratio, Gene: the nearest mapped gene.

**Table 2:** Clinical function annotation of mapped genes on chromosome 8.

Symbol	Chr	Start	End	Previous Relevant Studies
*LOXL2*	8	23154702	23282841	Severe Obesity and Obstructive Sleep Apnea
*ENTPD4*	8	23243296	23315208	Schizophrenia
*ADAMDEC1*	8	24241798	24263526	Attention‐deficit hyperactivity disorder
*ADAM7*	8	24298443	24384483	Melanoma
*NEFM*	8	24770525	24776607	Neurodegenerative disorders
*EBF2*	8	25699246	25902913	Neurogenesis
*BNIP3L*	8	26240414	26363152	Parkinson’s disease
*ADRA1A*	8	26605667	26724790	Depressive Disorder and Schizophrenia

We also attempted to replicate previous GWAS study reported sleep disorder associated variants^13,40^. Among reported significant SNPs, 5 SNPs (rs8180817, 7q31.1; rs7044885, 9q31.32; rs113851554, 2p14; rs12187443, 5q21.1; and rs701394, 5q14.1) showed significances between 3.50 × 10^−4^ and 9.70 × 10^−3^ in our samples (Table 1). In addition, 8 SNPs that showed suggestive significances in our study had marginal p values in previous studies^13,14^ (Supplementary Table 2).

GCTA was used to estimate the proportion of phenotypic variance explained by common SNPs. The common SNPs could explain 7% (se = 0.02, p = 0.015) of the phenotypic variability. This is consistent with several previous GWAS studies on insomnia^15,41^. Using GCTA, we also calculated the genetic correlation between insomnia and three substance use disorder phenotypes, namely alcohol (3,594 cases, 12,205 controls), nicotine (4,896 cases, 12,205 controls) and opioid (1,039 cases, 12,205 controls) use disorders, which were also extracted from the same study cohort using ICD codes (Supplementary Table 1). The strongest correlation was found between insomnia and alcohol use disorder (rG = 0.56, se=0.14, p < 0.001), followed by nicotine use disorder (rG = 0.50, se=0.12, p < 0.001) and opioid use disorder (rG = 0.43, se = 0.18, p = 0.02) (Table 3). Furthermore, we evaluated the genetic correlations between insomnia and a series of clinical conditions extracted from Partners Biobank using codified data. Among them, a moderate correlation was observed between insomnia and anxiety or type 2 diabetes (rG = 0.76, se = 0.38, p = 0.17; rG = 0.31, se = 0.14, p = 0.25) (Table 3). Limited by the sample size, we did not observe the significant correlations.

**Table 3:** Genetic correlation between insomnia and other clinical conditions.

Trait 1	Trait 2	SNP-based genetic correlation	SE	p-value
Insomnia	Alcohol use disorder	0.56	0.14	0.0001
Insomnia	Nicotine use disorder	0.50	0.12	0.0003
Insomnia	Opioid use disorder	0.43	0.18	0.0200
Insomnia	Anxiety	0.76	0.38	0.1900
Insomnia	Depression	0.72	0.70	0.3400
Insomnia	Bipolar	0.04	0.12	0.9200
Insomnia	Schizophrenia	0.22	0.31	0.4800
Insomnia	Type 2 Diabetes	0.31	0.14	0.2500

To gain more statistical power and further validate our results, we obtained the summary statistics from two recent insomnia GWAS, using data from UK Biobank or STARRS dataset^13,15^. We calculated the pair-wise genetic correlations between results from the Partners Biobank and these two studies and observed a moderate correlation between our results and Jansen *et al*. 2019 study (rG = 0.68, se    = 0.36, p = 0.18), while no significant correlation was found between Partners Biobank and Stein’s study (rG = 0.57, se = 1.28, p = 0.86). Lastly, a moderate correlation was observed between Jansen’ study and Stein’s study (rG = 0.35, se = 0.16, p = 0.07). We also checked the top two SNPs identified in Partners Biobank (rs17052966 and rs117915572) in both Stein’s and Jansen’s studies, but did not observe significant signals (STRASS: rs17052966: p = 0.95, beta = 0.002; rs117915572: p = 0.34, beta = 0.062; UKBB: rs17052966: p = 0.73, beta = 0.003; rs117915572: p = 0.83, beta = −0.002).

The meta-analysis was then conducted by combining our results from Partners Biobank and these two studies. Since the sample size of UK Biobank is significantly larger than our cohort and STARRS cohort, which can lead to a UK Biobank dominated meta-analysis result, we divided the meta-analysis into two steps: combining our results with the STARRS data alone (meta-1, N = 35,706) or combining all three studies (meta-2, N = 422,239) (Fig. 1b,c, Supplementary Fig. 1b,c, Supplementary Tables 3, 4). Two significant genomic loci were identified from meta-1 on chromosome 7 and 9, (Supplementary Tables 5 and 7). The leading SNPs, rs147549871 (p = 9.10 × 10^−9^) and rs7855172 (p = 1.32 × 10^−8^), from the identified loci were the top SNPs of the original study using the STARRS dataset. Also, the top SNPs rs17052966 and rs117915572 from Partners Biobank GWAS showed suggestive significances in the meta-1 analysis (p = 2.87 × 10^−5^ and p = 9.61 × 10^−6^). In meta-2 analysis, we identified 13 significant genomic loci with 31 independent significant SNPs, in which 11 loci were novel (Supplementary Tables 4 and 6). The top SNP, rs113851554 (p = 1.37 × 10^−21^), is on chromosome 2 and close to the *MEIS1* gene. *MEIS1* is a homeobox gene and plays an important role in neural crest development^42^. Multiple studies showed its relationship with sleep disorder, as well as restless legs syndrome (RLS)^13,14,19,43^.

In meta-analysis 2, using position mapping, we also identified 118 related genes within 10 kb region of significant SNPs. MAGMA tissue expression results suggested that genes from central nervous system tissues were highly enriched for expression (Supplementary Fig. 2). GWAS catalog analysis showed a series of previously reported sleep disorder genes, such as *MEIS1, CUL9 and FOXP2* (Supplementary Table 8)^40,44,45^.

## Discussion

Insomnia is one of the most prevalent mental disorders world-wide, affecting 10–20% of population. The strong genetic impact on insomnia has been repeatedly reported from different data sources. In many of these studies, self-reported insomnia symptoms were used to identify cases from the general population, which could limit our understanding of the complexity of this disease.

The current study used electronic health records and genomic information from a large patient cohort to conduct a GWAS on clinically defined insomnia phenotype. We discovered one novel genomic risk locus on chromosome 8. The leading SNP is in the region transcribes a long non-coding RNA, which has not been reported for insomnia. Differential expressions of several lncRNAs were shown to be associated with sleep deprivation^46^. In addition, among the eight genes mapped by our most highly significant SNP, 7 genes have been shown to be related with neuronal functions and psychiatric disorders, suggesting the possible significance of the genome region surrounding the discovered risk genomic locus.

We also conducted a large-scale meta-analysis by combining our results and 2 recent insomnia GWAS using data from UK Biobank and STARRS. The top identified SNP rs113851554 (p = 1.37 × 10^−21^) was among the top SNPs from Jansen *et al*. (p = 1.56 × 10^−51^) and Lane *et al*. (p = 9.76 × 10^−30^) 2019 studies^13,14^. Since the UKBB sample size is significantly larger than cohorts from Partners Biobank and STARRS, the result of meta-analysis was mainly driven by UKBB samples and the top SNPs from Partners GWAS did not show significances. However, we observed a moderated genetic correlation between study of Partners Biobank and Jansen’s report. Also, multiple significant SNPs we identified showed moderate significances in other GWAS, suggesting common components across them.

Substance use disorders, such as alcohol, nicotine and opioid, can also affect sleep patterns through various neurotransmitters and were shown to be significantly genetically associated with insomnia^47^. We found a strong positive genetic correlation between insomnia and these major substance use disorders among the same study population, providing more evidence for the relationship between psychiatric disorders and insomnia. Sleep patterns and multiple other clinical conditions were also showed to be closely connected. Studies have shown that sleep disorders affect more than 50% of adults with anxiety disorders^48^. Consistently, a moderate genetic correlation between insomnia and anxiety condition was observed in the current study. However, we did not observe significant correlations with depression, type 2 diabetes (we observed a moderate correlation) and schizophrenia which were previously reported^13,15^. Considering the previous correlation studies were mainly using summary statistics from UK Biobank, the different results we obtained could be caused by different definitions of these traits or the smaller sample size in our study.

Because of the broad definitions of insomnia, the phenotypes targeted by genome-wide association analysis have varied significantly across studies, ranging from primary insomnia to measurements of sleep length, sleep quality and early morning awakening. This could be one of the reasons for the fewer identified significant SNPs for insomnia and lack of consistent findings across studies. In this regard, electronic health records containing rich information about patient status and diagnostic information, can serve as an important data source of disease phenotypes.

This study has several limitations. First, insomnia is a common clinical symptom associated with multiple psychiatric disorders, which makes it very challenging to accurately define clinical insomnia. For the same reason, the genetic architecture identified by genome wide association studies can only reflect certain aspects of the complex insomnia phenotype. In this study, we used a simple ICD-code-based phenotype definition, and did not attempt to stratify the sample into multiple insomnia sub-phenotypes for GWAS due to the limitation of our sample size and the accuracy of the phenotyping method. We are planning to conduct following-up studies to further address these questions with larger sample size and other sources of phenotype information in the EHR, such as problem lists and clinical notes. Second, the study cohort is derived from a patient population, which could reflect more severe stage of insomnia. This could be one of the reasons we did not replicate several known insomnia related SNP from previous studies. Third, the cohort we extracted from Partners Biobank has a relatively small sample size compared with UK Biobank, which caused a significant imbalanced signal when conducting the meta-analysis.

In summary, we used clinical diagnosis information to identify insomnia cases among hospitalized patients. Our study cohort consists of clinically defined insomnia and provides a novel reference for insomnia genetic studies. Due to the heterogeneous clinical stages and complexity of the EHR data mining methods, we only utilized diagnostic codes in the development of our cohort in the current study. Based on this exploration, our developed pipeline will facilitate future research for more comprehensive genetic studies based on clinical records.

## Supplementary information

Supplementary information

Supplementary information 2

## Data Availability

The datasets generated and/or analyzed during the current study are not publicly available due to IRB regulations. The summary statistics are available from the corresponding author on request.
