# Supplementary material for: Genome-wide association analysis of insomnia using data from Partners Biobank
Source: Sci Rep. 2020 Apr 24;10:6928. doi: 10.1038/s41598-020-63792-0 (PMC7181749; doi:10.1038/s41598-020-63792-0)
Supplement: Supplementary file 1 — Supplementary information [file 41598_2020_63792_MOESM1_ESM.docx]

**Genome-wide association analysis of insomnia using data from Partners Biobank**

Wenyu Song^1, 2^, John Torous^3^, Joe, Kossowsky^4,5^, Chia-Yen Chen^6,7^, Hailiang Huang^6,7^, Adam Wright^1, 2, 8, 9^

^1^Departmet of Medicine, Brigham and Women’s Hospital, Harvard Medical School

^2^Department of Biomedical Informatics, Harvard Medical School

^3^Department of Psychiatry, Beth Israel Deaconess Medical Center, Harvard Medical School

^4^Department of Anesthesiology, Critical Care & Pain Medicine, Boston Children’s Hospital, Harvard Medical School

^5^Division of Clinical Psychology and Psychotherapy, University of Basel, Switzerland

^6^Psychiatric and Neurodevelopmental Genetics Unit, Analytic and Translational Genetics Unit, Massachusetts General Hospital, Harvard Medical School

^7^Stanley Center for Psychiatric Research, Broad Institute of MIT and Harvard

^8^Department of Biomedical Informatics, Vanderbilt University Medical Center

^9^Partners eCare, Partners HealthCare

**Supplementary Figure**

**Supplementary Figure 1. Q-Q plot of insomnia**

**a.** EHR based phenotype

**
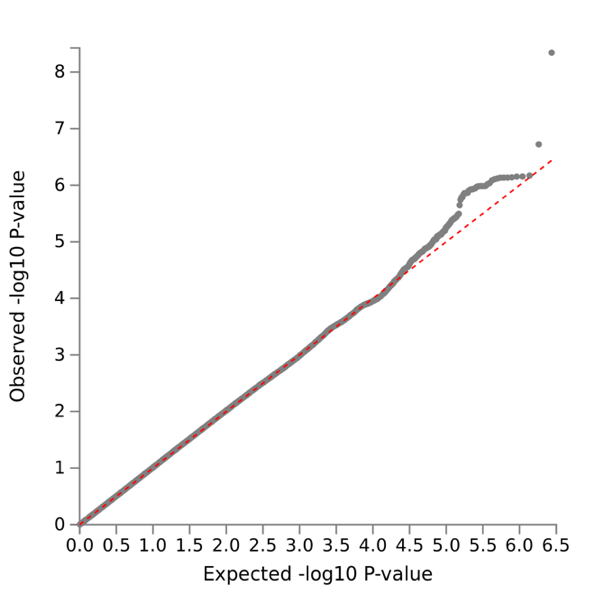
**

**b.** Meta-analysis 1

**
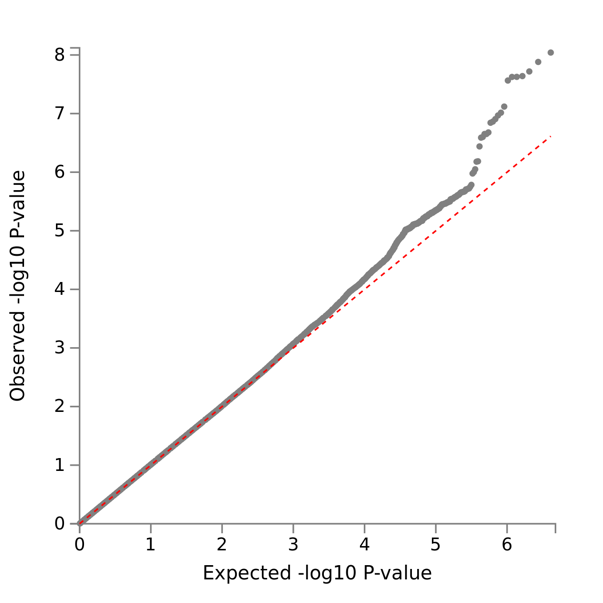
**

**c.** Meta-analysis 2

**
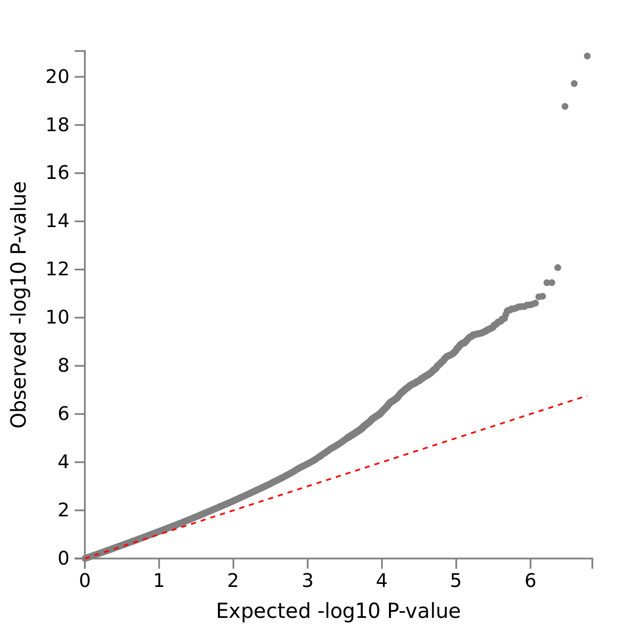
Supplementary Figure 2. Tissue expression profile analysis of meta-analysis2**

**
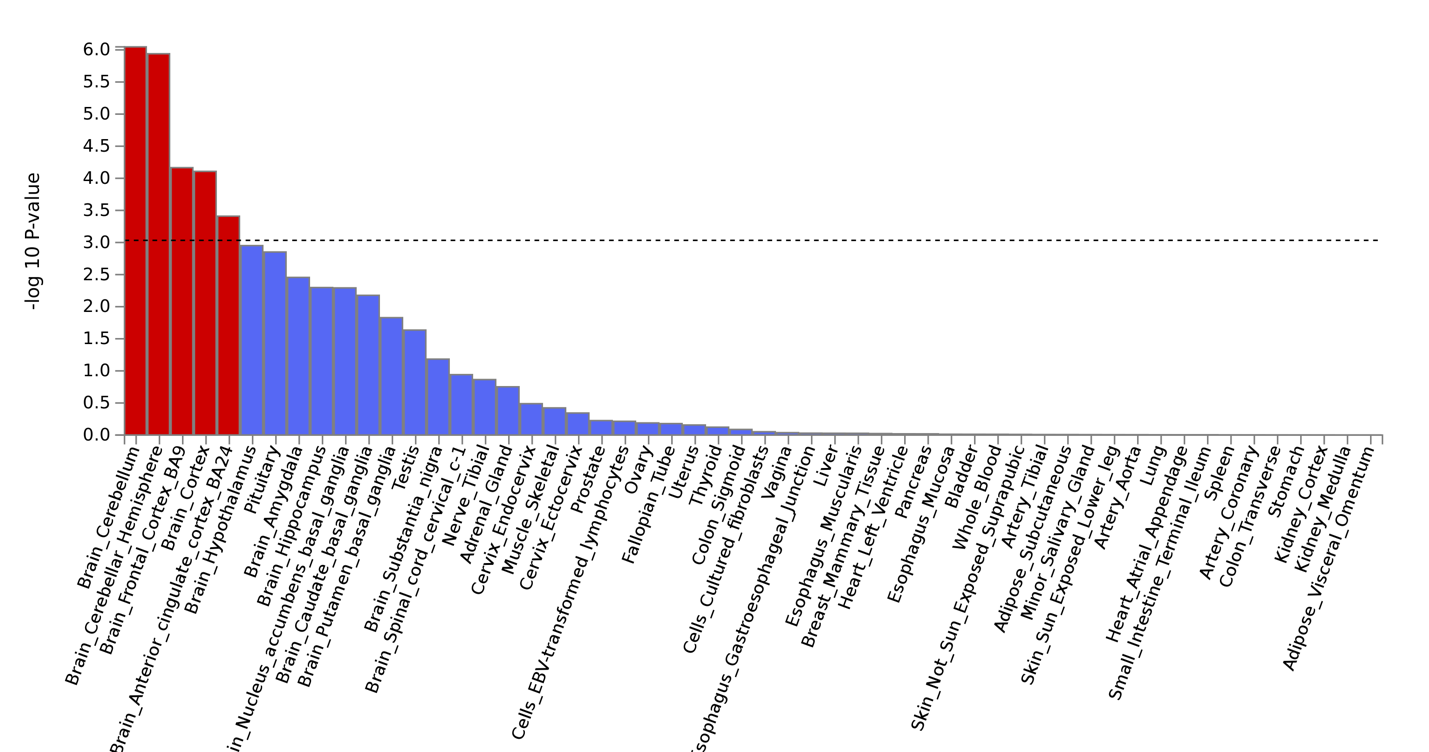
**
